# Cost-effectiveness and budget impact of breast cancer genetic profiling in Egypt

**DOI:** 10.3332/ecancer.2026.2101

**Published:** 2026-03-24

**Authors:** Amira Kotb, Wael Ali Hassan

**Affiliations:** 1 Department of Clinical and Chemical Pathology, Faculty of Medicine, Cairo University, Kasr Al-Ainy Street, Cairo, Postal Code 11562, Egypt; 2 Tawasol Holdings: 4 Shehab Street, Mohandessin, Giza, Postal Code 12655, Egypt; a https://orcid.org/0000-0002-0960-8202; b https://orcid.org/0000-0001-5998-9106

**Keywords:** cancer breast, genetic profiling, economic burden, BRCA1, BRCA2

## Abstract

Breast cancer is the most common cancer among women in Egypt and poses a growing public health challenge with significant socio-economic implications. While traditional diagnostic and treatment approaches remain central to care, advances in genetic testing offer new pathways for early detection, prevention and individualised treatment. Genetic testing, particularly for BRCA1, BRCA2 and other high-penetrance gene mutations, can identify women at elevated risk, enabling proactive surveillance, risk-reducing strategies and tailored therapeutic decisions. This article evaluates the cost-effectiveness of incorporating breast cancer genetic testing into national healthcare protocols. Evidence suggests that genetic screening for high-risk populations can significantly reduce long-term treatment costs by avoiding unnecessary interventions and improving survival through early detection and personalised therapy. In the Egyptian context, the upfront cost of testing can be offset by savings from more targeted chemotherapy, reduced recurrence rates and improved quality of life. The article further discusses the role of personalised medicine in transforming breast cancer care, emphasising the need for national policies that support equitable access to testing. It proposes practical guidelines, including risk-based screening criteria, integration of genetic counseling services, government-subsidised testing programs and capacity-building in local genomic laboratories. By adopting a structured, evidence-based approach to genetic testing and personalised care, Egypt can enhance treatment outcomes, reduce healthcare expenditures and align with global trends in precision oncology. The integration of genetic testing into routine oncology care not only supports clinical decision-making but also represents a strategic investment in the long-term sustainability and efficiency of Egypt’s healthcare system.

## Introduction

Breast cancer is the most frequently diagnosed cancer among women worldwide, with an estimated 2.3 million new cases and 670,000 deaths in 2022, accounting for 23.8% and 15.4% of all cancer cases and deaths in women, respectively (World Health Organisation, 2021). Projections indicate a substantial increase in both incidence and mortality by 2050, particularly in low- and middle-income countries (LMICs), due to factors such as population growth, aging and limited access to early detection and treatment [1].

In Egypt, breast cancer constituted approximately 17.8% of all newly diagnosed cancer cases among women, with an estimated 26,845 new cases in 2022 [2]. The 5-year prevalence reached 81,661 cases, representing 22.3% of all female cancer cases, underscoring its dominant burden within the national oncology landscape [40]. The age-adjusted incidence rate is estimated at 49.6 per 100,000 women, with a median age at diagnosis of 50–51 years, approximately a decade younger than the average age reported in Western populations [3].

Egypt's diagnostic protocol for breast cancer follows a structured, stepwise approach incorporating clinical, imaging and pathological assessments. The process typically begins with clinical breast examination (CBE), especially in primary care settings or general surgery clinics, often prompted by symptom presentation or participation in awareness campaigns [4]. For imaging, digital mammography remains the primary modality for women over 40, while ultrasound is commonly employed for younger women and those with dense breast tissue. In cases with inconclusive findings, contrast-enhanced mammography or breast magnetic resonance imaging may be utilised for enhanced diagnostic precision, particularly in high-risk or ambiguous cases [5]. The diagnostic pathway adheres to the triple test strategy, which combines physical examination, imaging and pathological assessment, improving diagnostic accuracy and clinical decision-making [5].

Suspicious lesions, particularly those classified as BI-RADS 4 or 5, are further evaluated through ultrasound-guided fine-needle aspiration biopsy (FNAB) or core needle biopsy, both of which are standard for histopathological confirmation [6]. In recent years, Egypt has implemented ‘one-stop’ breast clinics in partnership with Gustave Roussy and GE Healthcare, enabling patients to undergo clinical evaluation, imaging and biopsy in a single coordinated visit – thereby reducing diagnostic delays and improving cost-efficiency [7]. Despite these advances, systemic challenges such as fragmented referral pathways and limited awareness among general practitioners continue to contribute to delays in diagnosis and a high percentage of late-stage presentation [6]. Efforts to improve early detection and streamline the diagnostic process remain ongoing.

Egypt has undertaken several large-scale public health initiatives aimed at improving early detection, treatment access and overall awareness of breast cancer. The launch of the Women’s Health Outreach Program, in conjunction with the ‘100 Million Healthy Lives’ national project, marked a significant step in the country’s strategy to combat breast cancer. These programs have played a pivotal role in promoting early diagnosis, expanding access to free treatment service and enhancing awareness of women's health, particularly breast cancer. They also contributed to identifying comorbid conditions such as hypertension and diabetes, thereby facilitating more comprehensive healthcare delivery [7].

The projected rise in breast cancer incidence and mortality, particularly in LMICs like Egypt, is expected to significantly exacerbate the economic burden on already strained healthcare systems. Breast cancer not only imposes direct costs, including screening, diagnosis, treatment and palliative care, but also leads to substantial indirect costs due to lost productivity, long-term disability and premature mortality. In LMICs, where access to timely diagnosis and effective treatment remains limited, late-stage presentations are more common, leading to more intensive and expensive interventions and worse outcomes [8]. Between 2000 and 2019, cancer rose from the third to the second leading cause of death across nine countries in the Middle East and Africa, including Egypt, increasing from 10% to 13% of all deaths. It also became the third leading cause of disability-adjusted life years, up from sixth place, accounting for 8% of the total disease burden – up from 6%. During this period, new cancer cases per 100,000 people rose by 10%–100%. Looking ahead to 2040, demographic shifts alone are projected to drive further increases in cancer incidence, ranging from 27% in Egypt to 208% in the United Arab Emirates. In 2019, the economic burden of cancer varied widely across these countries, from around USD 15 per capita in the four African nations studied to USD 79 per capita in Kuwait [9].

The incorporation of genetic testing into breast cancer screening and diagnostic protocols offers significant advantages, particularly for individuals with a positive family history or other risk factors indicative of hereditary predisposition. Genetic profiling enables identification of mutations – such as BRCA1, BRCA2 and other susceptibility genes that not only reflect an inherited risk but also provide insight into tumour aggressiveness and potential response to specific therapies [10]. This precision medicine approach facilitates tailored treatment strategies, optimising therapeutic efficacy while minimising exposure to unnecessary or ineffective interventions, such as chemotherapy, thereby reducing treatment-related morbidity [11]. Consequently, genetic testing contributes to more cost-effective management by prioritising high-risk individuals for enhanced surveillance and personalised treatment, ultimately improving patient outcomes and generating broader public health benefits through resource allocation efficiency [12]. This strategy holds particular promise for healthcare systems in LMICs, such as Egypt, where optimised use of limited resources is critical for sustainable cancer control [13].

## Breast cancer: types, staging and therapeutic modalities

Breast cancer comprises a heterogeneous group of malignant neoplasms marked by uncontrolled proliferation, potential for local invasion and distant organ metastasis to sites such as the bones, lungs, liver and brain [14]. Histopathologically, tumours are categorised into *in situ* carcinomas ductal and lobular and invasive types. Among invasive forms, invasive ductal carcinoma (IDC) constitutes approximately 70%–80% of cases, while invasive lobular carcinoma (ILC) accounts for about 10%, with rarer variants including medullary, metaplastic, mucinous, tubular and neuroendocrine tumours [15].

Molecular classification, guided by gene expression profiling and immunohistochemical surrogates, identifies four principal intrinsic subtypes: Luminal A (ER+/PR+, HER2–, low Ki‑67), Luminal B (ER+, HER2–/+ with higher proliferation), HER2-enriched (ER–/PR–, HER2+) and Basal-like or triple-negative (ER–/PR–/HER2–) [15–17]. These molecular subtypes hold important prognostic value, with Luminal A associated with the most favorable outcomes, while HER2-enriched and basal-like tumours tend to demonstrate more aggressive behaviour and poorer survival rates. Additionally, pleomorphic variants of ILC often correspond to Luminal B or HER2 subtypes and are associated with worse outcomes [17].

Understanding these histological and molecular distinctions is essential for personalised treatment and prognostication. Such classification enables clinicians especially in resource limited settings to tailor therapies, improve patient stratification and optimise cost-effectiveness in breast cancer management [15].

The TNM classification system, developed by the American Joint Committee on Cancer and the Union for International Cancer Control, is the internationally accepted standard for staging breast cancer. It categorises disease progression based on three primary parameters: tumour size (T), regional lymph node involvement (N), and the presence of distant metastasis (M). In the eigth edition, the system was enhanced by the inclusion of key biological markers such as hormone receptor status (ER and PR), HER2 expression and tumour grade to define a prognostic stage. This integrative approach supports more accurate risk stratification and facilitates the application of personalised treatment strategies in clinical practice [18].

The treatment of breast cancer involves a multidisciplinary approach, with therapeutic decisions guided by an integrated assessment of tumour stage, biological characteristics, and patient-specific factors. Initial management typically includes surgical resection of the primary tumour, often accompanied by radiotherapy to reduce the risk of local recurrence or infiltration. Systemic therapies play a crucial role and include endocrine therapy for hormone receptor-positive disease, targeted therapy for HER2-positive subtypes, and chemotherapy when clinically indicated. Additionally, immunotherapy may be employed in selected cases of triple-negative breast cancer (TNBC). Recent advances in genomic testing have further refined personalised treatment strategies, enabling more precise risk stratification, improved clinical outcomes, and the avoidance of unnecessary toxicity and economic burden associated with overtreatment [19].

## Genetic basis of breast cancer

The genetic basis of breast cancer is well-documented, with hereditary mutations accounting for approximately 5%–10% of cases. The most significant genes associated with inherited breast cancer are BRCA1 and BRCA2, which are inherited in an autosomal dominant manner and demonstrate high penetrance, conferring lifetime breast cancer risks of 55%–72% and 45%–69%, respectively [20]. A study included 500 Egyptian women diagnosed with breast cancer (mean age: 47.3 ± 13.3 years) to evaluate the contribution of germline BRCA variants to breast cancer risk. Only HER2-negative patients were enrolled, while HER2-positive cases were excluded and managed with targeted therapy. A positive family history of breast and/or ovarian cancer was reported in 70.4% of participants. Pathogenic and likely pathogenic variants (PVs/LPVs) in BRCA1/2 were identified in 11.6% of patients, including 6.8% with BRCA1 and 4.8% with BRCA2 variants [21].

Other genes such as TP53, PALB2, PTEN, CHEK2, and ATM also contribute to hereditary risk, though with varying degrees of penetrance [22]. Having a first-degree relative affected by breast cancer approximately doubles an individual’s risk, with the risk further increasing depending on the number of affected relatives and the age at diagnosis within the family [23].

Genetic testing is essential for identifying individuals at elevated risk, particularly those with early-onset breast cancer, bilateral disease, or strong familial history. This testing supports preventive measures such as intensified screening, chemoprevention, or prophylactic surgery, and informs treatment options, most notably in BRCA mutation carriers who may benefit from PARP inhibitors like olaparib [24]. The adoption of multigene panel testing has expanded the detection of pathogenic variants beyond BRCA1/2, enhancing personalised care for both patients and their families [25].

In contrast, the vast majority of breast cancer cases approximately 85%–90% are sporadic, occurring in individuals without a hereditary mutation or notable family history. These cases arise mainly due to somatic mutations acquired over a person’s lifetime, influenced by environmental factors, hormonal exposures, aging, and random DNA replication errors. Frequently observed somatic alterations in sporadic breast cancers include mutations in TP53, PIK3CA, GATA3, and MAP3K1 [26].

Research exploring the genetic basis of breast cancer in Egypt. Has revealed a relatively high prevalence of BRCA1 and BRCA2 germline mutations, especially among high-risk groups, though nationwide data remain sparse. In a multicenter cohort study, Azim *et al* [27] reported that 14.8% of Egyptian breast cancer patients carried pathogenic BRCA1/2 mutations, and an additional 16.1% harbored variants of uncertain significance (VUS), indicating a substantial genetic contribution in this population. Another study conducted on Egyptian breast cancer patients and their first-degree female relatives aimed to detect germline mutations in selected exons of the BRCA1 and BRCA2 genes for early identification of presymptomatic carriers. The research involved 60 breast cancer patients from unrelated families and 120 healthy first-degree female relatives (sisters and/or daughters). Targeted PCR amplification was performed for BRCA1 exons 2, 8, 13, and 22 and BRCA2 exon 9, followed by SSCP, heteroduplex analysis, and DNA sequencing. Mutations in BRCA1 and BRCA2 were found in 86.7%of the families, with 60% of cases attributed to BRCA1 and 26.7% to BRCA2. Among healthy relatives, 67% (80/120) were identified as mutation carriers. Notably, BRCA mutations were observed in individuals both with and without a known family history of breast cancer, underscoring the importance of targeted genetic screening for early detection in high-risk populations [28]. In a survival study of 103 breast cancer patients over a 24-year follow-up, AbdelHamid *et al* [29] found that 28% were BRCA1/2 mutation carriers (29/103), and carrier status was associated with significantly worse recurrence-free survival, underscoring the clinical importance of early genetic identification. However, Kim et al. (2017) conducted whole-exome sequencing in five Egyptian families with a strong history of breast cancer and found no BRCA1/2 pathogenic variants, suggesting the presence of other, possibly population-specific, genetic susceptibility factors beyond BRCA in Egyptian cohorts [30]. These findings collectively highlight both the importance and the complexity of understanding the genetic landscape of breast cancer in Egypt.

## Genetic testing in breast cancer

### Types of genetic testing

Genetic testing for breast cancer includes a range of methods, each with specific applications and clinical relevance. Single-gene testing, traditionally focused on *BRCA1* and *BRCA2*, remains effective in families with a strong history of breast or ovarian cancer but offers limited scope. In contrast, multigene panel testing evaluates a broader set of genes – such as *TP53*, *PALB2*, *CHEK2* and *ATM* and improves mutation detection rates, though it may identify VUS [25]. SNP arrays and GWAS examine common low-risk variants like those near *FGFR2* and *TOX3*, contributing to emerging polygenic risk scores, though their current use is largely research-focused [31]. For patients with confirmed cancer, somatic tumour testing via next-generation sequencing identifies actionable mutations such as *PIK3CA* and *TP53*, supporting personalised therapy decisions, particularly in advanced disease [32]. Each testing modality plays a key role in refining risk assessment, guiding surveillance and optimising treatment strategies.

### Indications for genetic testing

Genetic testing has become an essential component of breast cancer management, facilitating risk assessment, early detection, treatment personalisation, and familial counseling. Current guidelines, particularly those from the National Comprehensive Cancer Network [14], recommend genetic testing for individuals with breast cancer diagnosed at age 45 or younger, and for those diagnosed before age 50 if they have additional risk factors such as a family history of related malignancies, bilateral breast cancer, or multiple primary cancers. Testing is also indicated in patients with TNBC diagnosed at or before the age of 60, as well as in all individuals with male breast cancer or a known familial pathogenic variant. In patients without a personal cancer history, a strong family history, such as a first degree relative diagnosed with breast cancer before age 50, ovarian cancer at any age, or a known familial mutation warrants consideration for testing. Beyond germline testing, tumour characteristics and molecular features such as homologous recombination deficiency or somatic *BRCA* mutations may also guide somatic testing, particularly when PARP inhibitors or other targeted therapies are being considered. Identifying pathogenic variants through genetic testing enables clinicians to tailor management strategies, recommend appropriate surveillance for at-risk family members, and consider risk-reducing interventions such as prophylactic mastectomy or oophorectomy (Assessment, n.d.).

### Genetic testing programs

Recent research highlights the growing support for expanding breast cancer risk assessment and genetic testing beyond traditional clinical criteria. Domchek and Robson [34] advocate for broader BRCA1/2 evaluation, noting that restrictive testing guidelines may overlook at-risk individuals without a strong family history. Supporting this, Hindmarch *et al* [35] demonstrate the feasibility and acceptability of offering breast cancer risk assessments to women aged 30–39 in the general population, suggesting earlier engagement in preventive strategies. Clinical guidelines summarised by Mcguire and Mamounas [36] emphasise the central role of genetic counseling and BRCA testing in managing hereditary breast cancer. Rainey *et al* [37] further show that personalised risk estimates can positively influence women’s health behaviours, promoting uptake of screening and lifestyle changes. Additionally, Torr *et al* [38] report the successful implementation of a population-based BRCA testing program in the Jewish community in England, highlighting its effectiveness in identifying mutation carriers and facilitating preventive action. Together, these findings underscore the potential of integrating both targeted and population-level genetic testing into routine breast cancer screening to enhance early detection and cost-effective prevention.

Despite the high prevalence of BRCA1/2 mutations reported in select Egyptian cohorts and the significant incidence and mortality of breast cancer among Egyptian women, the absence of a coordinated national cancer registry and a structured genetic screening program likely contributes to the underestimation of hereditary cancer risk, delayed diagnosis, potential overtreatment, and poorer clinical outcomes. The persistently high mortality rate further underscores the urgent need to implement a national, population-based genetic screening program to improve early detection and targeted prevention strategies.

## Cost-effectiveness of genetic testing

Breast cancer remains the most prevalent cancer globally. According to the World Health Organisation (2024) [59], an estimated 2.3 million new cases are diagnosed annually, comprising 11.6% of all new cancer cases. It is also a major cause of cancer-related mortality, accounting for around 670,000 deaths per year, or 6.9% of global cancer deaths. Although the exact global economic burden of cancer remains uncertain, projections suggest it will cost approximately $25.2 trillion globally between 2020 and 2050 [39].

In Egypt, cancer's economic burden was substantial in 2019, totaling $1.12 billion, or about $11 per capita. Direct healthcare costs contributed 44% of this total [9]. Breast cancer specifically accounts for 33% of all female cancer diagnoses in the country, with more than 22,000 new cases annually. While breast cancer was the leading cause of cancer-related deaths in Egypt in 2012 (29.1%), it became the second leading cause by 2018 (21.3%) [40].

In the broader Middle East and North Africa (MENA) region, cancer-related costs place significant strain on healthcare systems. In some MENA countries, cancer costs represent up to 0.8% of GDP, compared to 0.1%–0.2% in Gulf nations [9]. Delays in diagnosis and limited access to care exacerbate the financial burden, as treatment costs and outcomes are highly influenced by the stage at which cancer is diagnosed. Early-stage diagnoses (I and II) have survival rates ranging from 85% to 95%, while advanced-stage diagnoses (III and IV) result in survival rates between 30% and 70% [41].

In high-income countries like the United States, breast cancer patients incur significantly higher healthcare costs than those without cancer. Data from the Medical Expenditure Panel Survey between 2015 and 2018 show that 3.42% of nearly 466 million adult women were diagnosed with breast cancer, facing an annual incremental cost of $3,457 per person, 37% higher than that of non-cancer patients. Most of these costs ($3,237) were borne by payers, with out-of-pocket costs showing no significant difference [42].

In Serbia, the economic burden of breast cancer in 2019 amounted to approximately 15 million euros. Direct costs comprised 34% of the total, with hospital treatment making up 76% of this. Indirect costs, driven largely by productivity losses due to early retirement, represented 66% [43, 44].

Cancer's economic toll globally is substantial, reducing productivity, increasing unemployment, and limiting labor force participation. As such, improving access to early detection, screening, and treatment—particularly in LMICs—can yield significant economic and health benefits [44].

Costs associated with cancer care vary across different stages. According to the National Cancer Institute (2022) [60], the highest per-patient medical costs are seen in the end-of-life phase ($109,727), followed by the first year post-diagnosis ($43,516), with continuing care representing the lowest costs ($5,518). Prescription drug costs follow a similar pattern.

Given the financial strain, early detection and preventive strategies are critical, especially in LMICs. Breast cancer prevention efforts in Egypt primarily focus on early detection strategies and public education programsaimed at promoting healthy lifestyles and increasing screening participation. A central initiative is the Presidential Initiative for Supporting Egyptian Women’s Health, implemented under the broader ‘100 Million Healthy Lives’ campaign [45]. This nationwide, free program provides CBEs, along with prompt referral for diagnostic imaging, including mammography and ultrasound and access to treatment for confirmed cases through thousands of primary healthcare facilities.

Additional support is offered by non-governmental organisations, such as the Breast Cancer Foundation of Egypt [46], which deliver psychosocial support, rehabilitation services, and community-based awareness activities emphasising early detection practices, including breast self-examination and CBE. Evidence from academic studies demonstrates that these targeted educational interventions significantly enhance women’s awareness, screening behaviours, and adoption of preventive lifestyle practices, such as balanced nutrition and regular physical activity.

Thereby, acceptance of breast cancer prevention and screening interventions in Egypt demonstrates a clear contrast between the high participation rates achieved through national initiatives and the historically low engagement in routine individual screening practices. The Presidential Initiative for Supporting Egyptian Women’s Health, implemented by the Ministry of Health (MoH) and Population, has attained extensive nationwide coverage, reaching more than 70 million women through free clinical examinations and referral services for early tumour detection, underscoring the effectiveness of accessible, government-led screening programs in promoting initial uptake [45].

**Figure 1.**
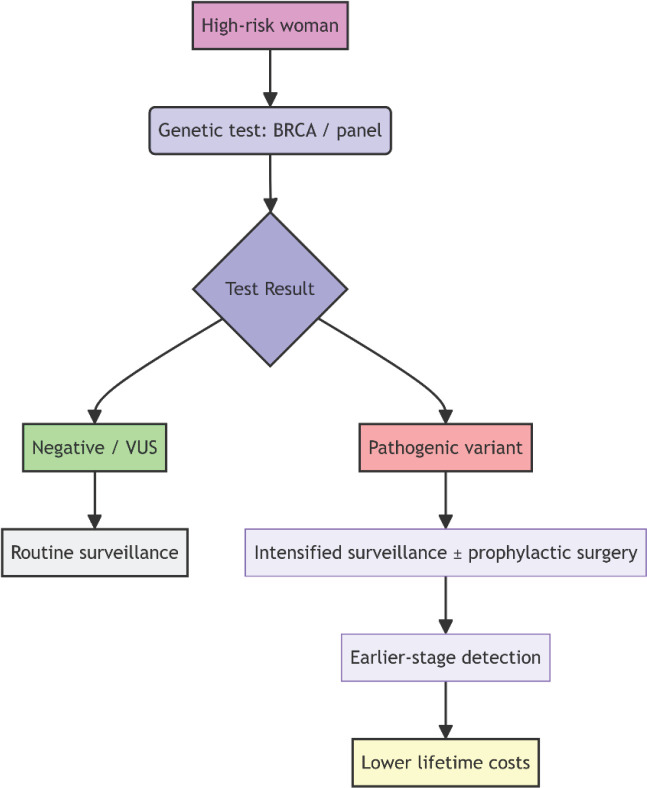
Conceptual pathway and economic effects of genetic profiling in Egypt (Conceptual pathway illustrating how genetic profiling for breast cancer in high-risk women leads to earlier-stage detection and reduced lifetime treatment costs).

Genetic testing for BRCA1 and BRCA2 mutations represents a cost-effective strategy for managing breast cancer (Figure 1). It enables risk stratification, early surveillance, and preventive interventions, which can reduce late-stage diagnoses, improve survival rates, and lower long-term healthcare costs. In resource-limited settings, where late diagnoses are common and treatment costs are high, BRCA testing could be a sustainable tool for cancer control. Integrating genetic testing into public health strategies could lead to more equitable and efficient cancer care in LMICs, reducing both clinical and financial burdens.

In Egypt, where cancer accounted for a significant economic burden of $1.12 billion in 2019 (about $11 per capita), the introduction of high-cost technologies like genetic profiling must be supported by comprehensive health economic evaluations (HEEs). Such evaluations will ensure that healthcare investments are both clinically effective and economically sustainable, particularly in the context of limited resources [47].

### HEE principles and their application to Egypt

#### Cost-effectiveness and cost-utility analysis (CUA)

HEE is a comparative analysis of alternative courses of action in terms of both their costs and their consequences (Table 1). A fundamental tool within HEE is the Cost-Effectiveness Analysis (CEA) and its more advanced form, the CUA. These methodologies produce a summary measure known as the Incremental Cost-Effectiveness Ratio (ICER), which compares the difference in costs between two interventions to the difference in their outcomes [48]. The formula for the ICER is expressed as:


$$\mathrm{ICER}\;=\;\frac{\mathrm{Cost}\;\mathrm{Intervention}-\mathrm{Cost}\;\mathrm{Comparator}}{\mathrm{Effectiveness}\;\mathrm{Intervention}-\mathrm{Effectiveness}\;\mathrm{Comparator}}$$

For this analysis, the primary outcome measure is the Quality-Adjusted Life Year (QALY), which is the standard metric used in CUA. QALYs are a comprehensive measure of health benefit because they account for both the quantity of life gained from an intervention (Life Years Gained) and the quality of life during those years, allowing for meaningful comparisons across different disease states and interventions. A secondary outcome measure is the Life Year Gained (LYG), which captures the extension of life without accounting for quality [48].

#### Cost-effectiveness thresholds

The interpretation of an ICER requires a benchmark known as the cost-effectiveness threshold or willingness-to-pay (WTP) threshold. This threshold represents the maximum amount a healthcare system is willing to pay to achieve one unit of health outcome, such as a QALY gained. The World Health Organisation (WHO) provides a widely used framework for LMICs, recommending a threshold of one to three times the country’s GDP per capita [49].

For Egypt, the GDP per capita in 2021 was $3,281. Following the WHO guidelines, a threshold of three times GDP per capita is applied, establishing a cost-effectiveness threshold of $9,843 per QALY for this analysis (World Economic Outlook (April 2025)). If an intervention's ICER falls below this threshold, it is considered a cost-effective use of limited healthcare resources.

#### Net monetary benefit (NMB) analysis

The NMB is an alternative and complementary metric to the ICER that expresses the net value of an intervention in monetary terms. This measure is particularly useful for decision-makers as it provides a clear, single figure indicating whether an intervention is economically favorable. The NMB is calculated by multiplying the health gain (in QALYs) by the cost-effectiveness threshold and then subtracting the incremental cost [50]. The formula is:

NMB = (Incremental QALYs × Threshold) − Incremental cost

A positive NMB signifies that an intervention's value, as defined by the system's willingness-to-pay, exceeds its cost, making it a sound economic investment. Evidence from a Colombian study of two genomic profiling tests demonstrated positive NMBs of $2,203 and $416, respectively [51].

#### Budget impact analysis (BIA)

While CEA and CUA provide a long-term perspective on the value of an intervention, BIAoffers a short-term, tactical view of its financial consequences for a specific payer, such as the Egyptian MoH. BIA is a critical tool for resource planning and addresses the fundamental question of affordability. It is distinct from CEA in that it focuses on direct costs and typically uses a short time horizon (1–5 years) without discounting, as decision-makers are concerned with immediate financial flows. A BIA models the expected changes in expenditure following the adoption of a new technology, considering factors like the target population size, the projected rate of uptake, and the costs and cost offsets of the intervention [52].

#### Opportunity cost considerations

Opportunity cost is a fundamental principle in economics, defined as the value of the next best alternative that must be forgone when a choice is made. In the context of a resource-constrained healthcare system like Egypt's, this concept is particularly relevant. The resources allocated to a new intervention, such as genetic profiling, are no longer available for other healthcare activities. The economic evaluation of an intervention must, therefore, assess whether the health improvement it offers exceeds the health improvement that could have been achieved by allocating those same resources to other interventions, such as broader population health programs. This necessitates a nuanced discussion that goes beyond a simple ICER calculation and evaluates the intervention within the broader context of national health priorities [53].

### Incremental analysis: calculating the ICER for genetic profiling in Egypt

#### Defining the incremental analysis

The core of this economic evaluation is the ICER, which compares the costs and outcomes of breast cancer genetic profiling against the existing standard of care (SOC) in Egypt.

- Intervention: Breast cancer genetic profiling, which may include targeted BRCA1/2 testing or more comprehensive multigene panels.
- Comparator: The existing diagnostic protocol in Egypt, which is a stepwise approach incorporating clinical, imaging, and pathological assessments.
- Effectiveness Measures: The primary outcome measure is QALYs gained. A secondary, but important, measure is LYG.

#### Incremental costs

The financial landscape of genetic testing in Egypt has undergone a dramatic transformation (Table 2). Current pricing for BRCA1/2 testing ranges from 16,609 to 20,000 EGP (approximately $530–$640 USD), while more comprehensive hereditary panels cost around 24,000 EGP ($770 USD). These prices represent a significant reduction from previous estimates of $3,551 per test [54]. This decline is critical to the economic viability of genetic profiling programs, as the initial investment directly impacts the incremental cost.

However, the incremental cost of the intervention is not simply the cost of the test itself. A complete analysis must consider the potential for cost savings, or *negative* incremental costs, over the patient's lifetime [55]. Genetic profiling can lead to avoided unnecessary treatments, as seen in a Colombian study where genomic profiling tests generated cost savings of $554–$2,374 compared to standard chemotherapy-for-all approaches [51]. By identifying high-risk individuals, genetic testing can also enable earlier, less expensive cancer treatments and reduce diagnostic workup costs, thereby offsetting the initial investment.

#### Incremental outcomes

The primary measure of effectiveness is the number of QALYs gained from the intervention compared to the SOC. A Colombian study found that genomic profiling tests increased QALYs by 0.03–0.05 [51]. While this may appear to be a small numerical value, it represents a substantial health gain when extrapolated across a large population over a lifetime horizon. The provided research material does not contain specific data on Incremental Life Years Gained, but this metric is a standard component of comprehensive health economic models and would be included in a full-scale analysis.

#### Calculation and assessment of ICER

**Figure 2.**
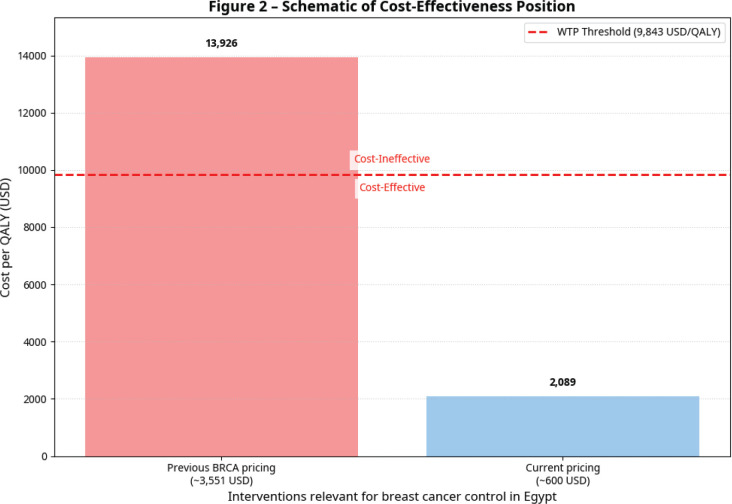
Schematic of cost-effectiveness position.

The estimated ICER for breast cancer genetic profiling in Egypt is derived from a key data point: the previous ICER of $13,926 per QALY gained (Figure 2). This figure was based on an earlier, higher testing cost. The dramatic reduction in the cost of genetic testing, by a factor of approximately 85% (from $3,551 to an average of $600), fundamentally alters this calculation. By applying a proportional reduction to the previous ICER, the revised estimated ICER is approximately $2,089 per QALY gained.

This is a profound and critical shift in the economic profile of the intervention. The previous ICER of $13,926 would have placed genetic testing outside of Egypt's cost-effectiveness threshold of $9,843 per QALY, likely deeming it an inefficient use of resources. However, the revised estimated ICER of $2,089 per QALY falls well below this threshold, indicating that genetic profiling is a highly cost-effective intervention by WHO standards. This demonstrates that the global trend of technology commoditisation, particularly the significant reduction in the cost of genomic sequencing, serves as a powerful strategic lever for LMICs, enabling them to implement advanced, formerly unaffordable healthcare solutions.

#### NMB analysis

The NMB analysis provides a complementary perspective to the ICER, expressing the economic value of an intervention as a single monetary figure. This is particularly valuable for decision-makers who may find it easier to interpret a net financial gain or loss [56]. A positive NMB indicates that the value of the health benefits generated by the intervention outweighs its costs at the specified WTP threshold.

International evidence provides a tangible example of this concept. A Colombian study on two genomic profiling tests, Oncotype DX and MammaScript, found that they generated positive NMBs of $2,203 and $416, respectively [51]. This demonstrates that even after accounting for the costs, these interventions represented a net financial gain for the healthcare system.

In the Egyptian context, the estimated ICER of approximately $2,089 per QALY gained is substantially lower than the country's cost-effectiveness threshold of $9,843 per QALY. This favorable ratio guarantees a positive NMB. Using the conceptual NMB formula, the value of the QALYs gained from genetic profiling, when multiplied by the threshold, will significantly exceed the incremental cost of the intervention. This reinforces the conclusion from the ICER analysis: genetic profiling is not only clinically beneficial but also an economically advantageous investment for the Egyptian healthcare system.

#### BIA and financial implications

While the long-term cost-effectiveness of genetic profiling is well-supported by the ICER analysis, a critical question for the Egyptian MoH is the short-term financial feasibility or affordability of a national program. A BIA is essential for answering this question. 

A conceptual BIA framework for the Egyptian MoH must consider several key factors. First, the target population must be defined, whether it is all new breast cancer patients, high-risk women with a strong family history, or a combination. Second, a phased implementation strategy is a crucial recommendation from the provided documents, which would involve a gradual rollout of the program, for example, starting with a 5% uptake in the first year and expanding incrementally. This approach spreads the initial capital outlay over time, making it a more manageable expense for a resource-constrained budget.

The BIA must account for the primary cost drivers, including the acquisition cost of the genetic tests ($530–$770 per test) and the associated costs of genetic counseling and intensified surveillance for high-risk individuals. However, it must also quantify the significant cost offsets that can be realised.

The breast cancer screening program in an Egyptian slum, where screened patients had average lifetime treatment costs of $28,632, which was 50% lower than the $58,170 for unscreened patients [57]. This finding is highly relevant, as it demonstrates that early detection, facilitated by genetic profiling, can lead to substantial long-term savings by avoiding the high costs of treating advanced-stage cancer, which includes expensive drug therapies, hospital stays, and end-of-life care.

This analysis highlights a critical paradox: while genetic profiling is cost-effective over a lifetime horizon, the initial capital outlay for a large-scale program could represent a significant budget shock. The BIA serves as the tool to bridge the gap between long-term value and short-term affordability. The phased implementation strategy directly addresses this challenge, allowing the MoH to gradually build the necessary infrastructure—including laboratory capacity, workforce training, and policy development—while the accrued cost savings from averted late-stage treatments provide a financial offset over time.

#### Opportunity cost: balancing genetic profiling with broader population health interventions

In a healthcare system with finite resources, every investment decision carries an opportunity cost. The economic evaluation of an intervention must, therefore, be framed not only in terms of its direct value but also in comparison to the value of other potential investments [58].

A community-based breast cancer screening program in an Egyptian slum demonstrated an extraordinary 133% return on investment for facility-based screening [57]. This finding introduces a crucial policy question: Is it more beneficial to allocate resources to a high-cost, high-yield intervention for a specific, high-risk cohort (genetic profiling) or to a lower-cost, broader intervention with a potentially greater overall health impact?

The ICER analysis, which compares genetic profiling to a ‘no testing’ scenario, provides a limited view of this complex trade-off. The real policy consideration is whether the ICER of ~$2,089 per QALY is more or less favorable than the ICER of other interventions available to the MoH. The high return on investment from a simpler, population-wide screening program suggests that policymakers must carefully weigh the value of genetic profiling against other, potentially more impactful, public health investments. This elevates the analysis from a simple cost-effectiveness calculation to a nuanced discussion of national healthcare priorities.

A study on an Egyptian cohort found that 67% of healthy first-degree relatives of affected patients were mutation carriers [28]. This remarkably high yield means that the economic and health value of a genetic profiling program is not limited to the index patient. Through cascade testing, the intervention can identify a large number of asymptomatic, high-risk individuals and facilitate preventive measures, thereby averting future cases of cancer and the associated costs. This high-yield characteristic of cascade testing blurs the line between a targeted clinical intervention and a broader population screening tool, suggesting that the most effective strategy for Egypt is a synergistic one: integrating genetic profiling into existing public health initiatives to maximise overall health benefits.

## Discussion, recommendations, and future research directions

### Synthesis of economic evidence

The economic evidence strongly supports the integration of breast cancer genetic profiling into the Egyptian healthcare system. The dramatic reduction in the cost of genetic testing has fundamentally altered its economic profile, making it a highly cost-effective intervention with an estimated ICER of approximately $2,089 per QALY gained. This value falls well below the country's cost-effectiveness threshold of $9,843 per QALY, demonstrating that genetic profiling represents a sound investment. Furthermore, the analysis indicates a strong potential for a positive NMB and long-term cost savings by shifting the focus from expensive late-stage treatment to prevention and early detection.

The economic case is further strengthened by the unique epidemiological and demographic factors of the Egyptian population, including a younger median age at diagnosis and large family structures. The high prevalence of BRCA mutations in certain cohorts [29], coupled with the potential for high-yield cascade testing, transforms genetic profiling from a niche, high-cost intervention into a powerful public health tool with population-level benefits.

## Strategic recommendations for the Egyptian MoH.

Based on the evidence and analysis, the following strategic recommendations are proposed to the Egyptian MoH:

- **Phased implementation:** A full-scale, nationwide program should be avoided in favor of a strategic, phased rollout. The initial phase should focus on pilot programs targeting high-risk populations and establishing genetic testing services in key cancer centers. This approach mitigates the short-term budget impact and allows for the gradual development of necessary infrastructure.
- **Policy and reimbursement frameworks:** The MoH should work with relevant stakeholders to establish clear national reimbursement mechanisms and clinical guidelines. These guidelines should define the indications for genetic testing and ensure standardised, equitable access for all eligible patients.

## Critical research gaps and future directions

The most significant limitation of the current analysis is the reliance on data from international studies and models to estimate the ICER for the Egyptian context. To ensure that policy decisions are based on the most accurate and relevant information, there is an urgent need for the following research to be conducted:

- **Formal BIA:** A formal BIA for the Egyptian healthcare system is a priority need. This analysis would model the precise short-term financial implications of a phased implementation strategy, providing policymakers with a clear roadmap for resource planning.
- **Long-term outcome tracking:** There is a need for studies that track the long-term clinical and economic outcomes of genetic testing recipients in Egypt. This would provide real-world data on the impact of testing on treatment choices, survival rates, and lifetime healthcare costs.
- **Assessment of cascade testing benefits:** Research should be conducted to formally assess the full economic benefits of family cascade testing in the Egyptian context, modeling the population-level health gains and cost savings from preventing future cancers in at-risk family members.
- **National cancer registry:** The establishment of a coordinated national cancer registry is a foundational requirement for all future HEEs. A registry would provide the robust, population-specific data necessary to enable a truly data-driven approach to health policy and resource allocation.

## Conclusion

The accumulated clinical and economic evidence supports the phased implementation of breast cancer genetic profiling within the Egyptian healthcare system. Although national efforts such as the Women’s Health Initiative have strengthened early detection, access to advanced genetic testing remains limited due to cost barriers and uneven availability, resulting in persistent disparities in care. Recent reductions in testing costs, however, have fundamentally altered the economic landscape, transforming genetic profiling from a prohibitively expensive technology into a cost-effective and economically sustainable intervention.

From a health system perspective, the initial budgetary impact of introducing genetic profiling is offset by its long-term economic and public health benefits. Egyptian women are frequently diagnosed at younger ages and with more advanced disease, leading to poorer outcomes and higher treatment costs. Genetic profiling enables the early identification of individuals at high hereditary risk, supporting targeted prevention strategies, intensified surveillance, and cascade testing among relatives. These approaches have the potential to avert costly late-stage treatments, improve survival, and generate substantial downstream cost savings.

A structured national program, beginning with high-risk populations defined by clinical and family history criteria, offers a pragmatic and equitable pathway for implementation. Strategic collaboration between the MoH and private-sector stakeholders, combined with public education initiatives and the ongoing expansion of the Universal Health Insurance System, provides a realistic framework for scaling access to personalised cancer care. Ultimately, integrating genetic profiling into breast cancer management represents not only a meaningful clinical advance for Egyptian women but also a strategic investment in prevention, health system efficiency, and the long-term sustainability of Egypt’s healthcare sector.

## Conflicts of interest

The authors have no relevant financial or non-financial interests to disclose.

## Funding

The authors declare that no funds, grants, or other support were received during the preparation of this manuscript.

## Figures and Tables

**Table 1.** Summary of key economic indicators used in the model.

Parameter	Base value	Source/Rationale
GDP per capita (USD)	3,281	World Economic Outlook 2025
WTP threshold (USD/QALY)	9,843	3× GDP per capita (Standard WHO-CHOICE recommendation for cost-effectiveness)
Average BRCA test cost (USD)	600	Midpoint of the current pricing range (530–640 USD)
Previous BRCA test cost (USD)	3,551	Historical pricing used in the initial CEA
ICER at previous cost (USD/QALY)	13,926	Model result at the historical test price (Above WTP threshold)
ICER at current cost (USD/QALY)	2,089	Model result at the updated test price (Well below WTP threshold)
Lifetime cost for screened women (USD)	28,632	Model output: Cost of care for high-risk women identified and managed with prevention/early detection.
Lifetime cost for unscreened women (USD)	58,170	Model output: Cost of care for high-risk women managed without genetic screening (often leading to late-stage diagnosis).

**Table 2.** Types of genetic testing and indicative prices in Egypt.

Test type	Genes included	Clinical indication	Approximate price (EGP/USD)	Main economic implication
**BRCA1/2 targeted sequencing**	*BRCA1*, *BRCA2*	High-risk individuals, strong family history, early-onset breast/ovarian cancer.	16,609–20,000 EGP	Enables risk-reducing strategies (surveillance/surgery); **Cost-effective at current pricing.**
**85-gene hereditary panel**	*BRCA1*, *BRCA2*, *PALB2*, *CHEK2*, *ATM*, *TP53*, etc. (85 genes)	Broader hereditary cancer risk assessment, especially when *BRCA* is negative or for complex family histories.	24,000 EGP	Identifies additional high-risk individuals for prevention; Higher upfront cost, but potentially higher yield.
**Somatic NGS panel**	Varies (e.g., 50–500 genes)	Advanced/metastatic disease; guiding targeted therapy (e.g., PARP inhibitors, PIK3CA inhibitors).	Contextual only (Price not specified in text)	Guides treatment selection, avoiding ineffective chemotherapy; **Reduces downstream treatment costs.**
